# Beyond Digital Therapy: Augmenting Internet-Based Cognitive Behavioral Therapy With Brain Stimulation

**DOI:** 10.1016/j.bpsgos.2025.100514

**Published:** 2025-05-05

**Authors:** Willians F. Vieira

**Affiliations:** Department of Structural and Functional Biology, Institute of Biology, University of Campinas (UNICAMP), Campinas, Brazil

In the new study by Barrett *et al.* (1) in *Biological Psychiatry: Global Open Science*, the investigators explore an innovative approach to enhancing cognitive behavioral therapy (CBT) for major depressive disorder (MDD) by incorporating transcranial infrared laser stimulation (TILS). This noninvasive neuromodulation technique, which facilitates prefrontal cortex energy metabolism and oxygenation (2,3), is evaluated as an adjunct to internet-based CBT (deprexis). The findings suggest that TILS significantly augments the antidepressant effects of CBT, presenting a potential breakthrough in the treatment of depression.

One of the notable strengths of this study is its rigorous experimental design. The researchers used a randomized, placebo-controlled methodology, which enhances the validity of their findings. By assigning participants who showed initial improvement with deprexis by at least 10% after 2 weeks to either TILS or placebo groups, the study ensured that only participants who were responsive to the intervention were included in the neurostimulation phase. This approach minimizes the effect of confounding variables and strengthens the causal inference that TILS contributes to improved outcomes. The placebo condition involved applying the laser to the forehead for 5 seconds followed by 55 seconds with the laser turned off, during each 1-minute cycle. This represents just one twelfth of the energy that was delivered to the experimental group, and according to the authors, it did not produce any cognitive effects.

Another strength lies in the use of a well-validated self-report measure, the Quick Inventory of Depressive Symptomatology–Self-Report (QIDS-SR), to assess changes in depressive symptoms. The repeated-measures analysis over the 12-week period provides a comprehensive view of symptom trajectory, confirming the sustained efficacy of the intervention. QIDS-SR scores decreased by 43% in the sham group from baseline to week 12, whereas the addition of TILS as an adjunct therapy led to a 56% reduction. This corresponds to an additional 30% improvement in QIDS-SR scores with TILS. Furthermore, the study’s focus on a noninvasive neuromodulation technique is particularly relevant given the need for safer, more accessible, cost-effective, and self-administered alternatives to traditional treatments such as pharmacotherapy and electroconvulsive therapy. TILS’s potential to enhance mitochondrial energy metabolism and modulate neural activity (2,3) is consistent with emerging research on bioenergetic deficits in depression, providing a compelling mechanistic explanation for its observed effects. Such effects of TILS result from the ability of near-infrared and red light to penetrate brain tissue, where the energy is absorbed by specific cellular chromophores, leading to biological and therapeutic effects (4,5).

Despite its strengths, the study has some limitations that warrant discussion. First, the sample size, while adequate for detecting statistical significance, remains relatively small (*n* = 40 for the randomized phase). Given the heterogeneity of MDD, larger and more diverse cohorts are needed to ensure the generalizability of these findings across different populations. Second, the study exclusively targeted individuals who showed an initial response to deprexis. While this design choice was intentional, it raises questions about whether TILS would benefit individuals who do not initially respond to internet-based CBT. Future studies should explore whether TILS can serve as an effective standalone intervention or whether it requires synergy with cognitive interventions to yield significant results. Another limitation concerns the reliance on self-reported outcomes. While the QIDS-SR is a validated instrument, subjective measures can introduce biases such as social desirability or response expectancy effects. The inclusion of neuroimaging or neurophysiological assessments might have strengthened the study by providing objective biomarkers of the impact of TILS on brain function. Additionally, while the authors report no adverse effects of TILS, the study’s follow-up period was limited to 12 weeks. Long-term safety and efficacy data are necessary to establish TILS as a viable clinical tool. Questions remain about whether the benefits persist beyond the study period and whether repeated TILS sessions are required to maintain therapeutic effects.

Barrett *et al.*’s (1) findings contribute to a growing body of literature on neuromodulation as an adjunct to psychotherapeutic interventions. The observed synergy between TILS and CBT suggests a promising direction for integrating biological and psychological treatment modalities. A recent systematic review by Farazi *et al.* (6) explored the TILS combination therapy as a new insight in neurological disorders, including MDD. They mentioned the studies conducted by Zaizar *et al.* (7,8), which reported that the combined application of TILS and exposure therapy effectively alleviated pathological fear. Additionally, research findings have indicated that integrating TILS with a static magnetic field and Pilates, which is a recognized therapeutic strategy for stress incontinence, enhances muscle strength and minimizes urinary leakage. Moreover, certain investigations have suggested that pairing TILS with pharmaceutical agents, such as coenzyme Q10, mitigates anxiety by counteracting oxidative stress, neuroinflammation, and neuronal apoptosis (9). Furthermore, the simultaneous use of transcranial photobiomodulation and an enriched environment has been associated with increased hippocampal levels of BDNF (brain-derived neurotrophic factor), elevated TrkB expression, and an improved p-CREB/CREB (cAMP response element binding protein) ratio, together with a reduction in depressive behaviors (10).

Future research should focus on replicating these findings in larger, more diverse samples and exploring individual differences in response to TILS. It would also be valuable to compare TILS with other forms of neuromodulation, such as transcranial magnetic stimulation or transcranial direct current stimulation, to determine its relative efficacy and cost-effectiveness. Moreover, mechanistic studies that use neuroimaging techniques such as functional magnetic resonance imaging or positron emission tomography scans could elucidate how TILS influences neural circuits that are implicated in depression. Understanding whether TILS primarily affects emotion regulation, cognitive control, or neuroplasticity could refine its application in clinical practice.

Barrett *et al.* (1) present compelling evidence that TILS can enhance the therapeutic effects of internet-based CBT for MDD. Their study underscores the potential of combining neuromodulation with psychotherapeutic approaches to improve treatment outcomes. However, additional research is needed to confirm these findings, explore long-term effects, and refine the clinical application of TILS. If validated, this approach could represent a significant advancement in the personalized treatment of depression, offering a noninvasive, accessible, and effective therapeutic option for individuals struggling with this debilitating disorder.
